# Targeting the RBD of Omicron Variant (B.1.1.529) with Medicinal Phytocompounds to Abrogate the Binding of Spike Glycoprotein with the hACE2 Using Computational Molecular Search and Simulation Approach

**DOI:** 10.3390/biology11020258

**Published:** 2022-02-07

**Authors:** Abdulrahim R. Hakami

**Affiliations:** Department of Clinical Laboratory Sciences, College of Applied Medical Sciences, King Khalid University, Abha 61481, Saudi Arabia; ahakami@kku.edu.sa; Tel.: +966-(0)17241-9268

**Keywords:** SARS-CoV-2, Omicron, medicinal plants, bioinformatics, MD simulations

## Abstract

**Simple Summary:**

The current study based on virtual drugs screening and simulations identified novel drugs to target the RBD of the spike protein from Omicron variant of SARS-CoV-2. Using molecular modeling tools to search for a good binding drugs we identified SANC00944, SANC01032, SANC00992, and SANC00317 from South African natural compounds database as potential inhibitor of the Spike-ACE2 complex. In sum, this study will help in the design and discovery of novel drug therapeutics, which may be used against the emerging Omicron variant of SARS-CoV-2.

**Abstract:**

The severe acute respiratory syndrome coronavirus 2 (SARS-CoV-2) virus continues to inflict chaos globally. The emergence of a novel Omicron variant (B.1.1.529) in South Africa harbors 30 mutations in the spike protein. The variant is distinguished from other variants of concern (VOCs) with an increased (15) number of mutations in the receptor-binding domain (RBD) and suggests higher chances of causing reinfections. Initial reports also claimed that this variant escapes all the neutralizing antibodies, thus demanding a novel strategy against it. Thus, in this study, we performed a computational molecular screening against the RBD of the Omicron (B.1.1.529) variant and assessed the binding affinity of potent drugs against the RBD. The multi-steps screening of the South African Natural Compounds Database (SANCDB) revealed four medicinal compounds as excellent (potential) anti-viral agents against the Omicron variant, namely SANC00944, SANC01032, SANC00992, and SANC00317. The simulation analysis of these compounds in complex with the RBD demonstrated stable dynamics and structural compactness. Moreover, the residual flexibility analysis revealed that the flexibility of three loops required for interaction with hACE2 has been reduced by the binding of these drugs. The post-simulation validation of these compounds such as binding free energy, in silico bioactivity, and dissociation constant prediction validated the anti-viral potency of these compounds. The total binding free energy (TBFE) for the SANC01032–RBD complex was reported to be −46.54 kcal/mol; for the SANC01032–RBD complex, the TBFE was −41.88 kcal/mol; for the SANC00992–RBD complex the TBFE was −29.05 kcal/mol, while for the SANC00317–RBD complex the TBFE was −31.03 kcal/mol. The results showed the inhibition potential of these compounds by targeting the RBD. In conclusion, this study will help in the design and discovery of novel drug therapeutics, which may be used against the emerging Omicron variant of SARS-CoV-2.

## 1. Introduction

**Figure 1:**
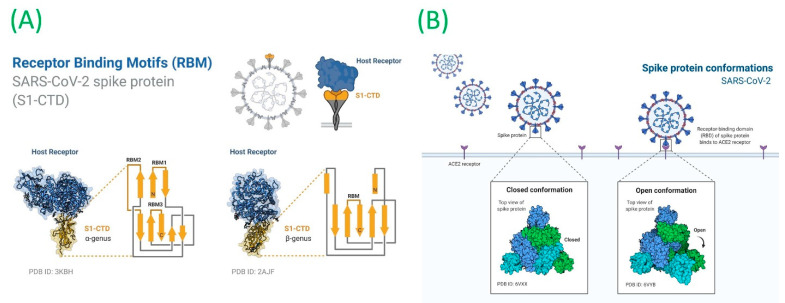
(**A**) Receptor-binding motifs of the severe acute respiratory syndrome coronavirus 2 (SARS-CoV-2) spike protein and its conformation. (**B**) Binding of spike and ACE2. Images were created using BioRender.com (accessed on 16 January 2022).

Severe acute respiratory syndrome coronavirus 2 (SARS-CoV-2) continues to cause a major public health burden. This is reflected by the high prevalence of the SARS-CoV-2-associated coronavirus disease (COVID-19) around the globe [1,2]. Despite the expedited vaccination to combat against the SARS-CoV-2, the continued emergence of new variants might out-compete the efficacy of already developed vaccines [3,4]. This includes three different categories of SARS-CoV-2 variants classified as variants of interest (VOIs), variants of concern (VOCs), and variants of high consequence (VOHCs) [5]. Among these, highly transmissible VOCs have been specifically linked with severe disease outcomes, reduced antibody neutralization, and poor treatment response [6]. These VOCs mainly harbor mutations in the receptor-binding domain (RBD) of the spike (S) protein that is mainly composed of two subunits [7,8]. Among these, subunit 1 (S1) contains the host ACE2 RBD, while subunit 2 (S2) contributes as a whole to the fusion of the RBD with the host hACE2 (Figure 1A). Moreover, RBD-associated mutations confer conformational changes in its structure (Figure 1B) and are linked with higher rates of reinfection and adapted mechanisms to evade the host immunity [9,10,11].

Many variants of SARS-CoV-2 that emerged with distinct mutations particularly on the RBD of spike proteins are reported. Among the VOCs, delta (δ) plus or B.1.617.2.1 acquires K417N, L452R, and T478K mutations in the RDB. The impact of these mutations has been reported by a study using a structural approach and revealed that these mutations increase the binding affinity for the hACE2 [12]. Similarly, the δ variant and B.1.621 (Mu) are also reported to exhibit several shared mutations with other VOCs, i.e., E484K, N501Y, and P681H. Other mutations, i.e., R346K, Y144T, Y145S, and 146N insertion, have been reported. The lineage B.1.1.1, which harbors L452Q and F490S mutations in the RBD, has been characterized to escape the antibody neutralization. Similarly, the kappa (κ) variant carries mutation L452R and is associated with altered antibody neutralization by disrupting the respective conformational epitopes. The other variant with E484K mutation also confers resistance to antibodies [12,13,14,15]. Recently, a newly emerged Omicron variant (B.1.1.529) in South Africa harbors 30 mutations in the spike protein. The variant is distinguished from other VOCs with an increased (15) number of mutations in the RBD and suggests higher chances of causing reinfections [16]. The most prevalent cases of this variant were found common in several provinces of South Africa. The variant poses a significant health threat around the world, and the therapeutic efficacy of already developed vaccine therapeutics against it remains elusive [17,18]. Further investigations are required to depict the molecular mechanism of pathogenicity related to the Omicron variant. Moreover, improved strategies are needed to design novel therapeutics against the newly emerged SARS-CoV-2 variants.

The challenging SARS-CoV-2 pandemic conditions urgently require the development of safe and efficient treatments options [19,20]. Herein, the spike protein is considered as the most potent target in therapeutics design against the corona disease [21,22]. Specifically, the increased occurrence of mutations in the RBD of SARS-CoV-2 related to increased viral affinity towards host receptors and higher infectivity is critical to be evaluated as a drug target. In this study, we performed virtual screening against the Omicron (B.1.1.529) RBD and assessed the binding affinity of potent drugs against the RBD. The present work involved in silico methods including the molecular screening of the South African Natural Compounds Database (SANCDB), the simulation investigation of the top, and binding free energy calculations. The results demonstrated critical information about the anti-viral potency of the screened drugs against the mutations in the RBD of the Omicron variant. The study will help in the design and discovery of novel drug therapeutics, which may be deployed against the emerging Omicron variant of SARS-CoV-2.

## 2. Materials and Methods

### 2.1. Structure Retrieval and Modeling

A comparative modeling approach was used to model the 3-dimentional structure of the RDB of the Omicron variant by using Modeler embedded in Chimera Software [23,24,25]. A crystallized structure of the wild-type RBD with the Protein Data Bank ID of 6M0J was used as a template, because it shares a 95% identity with the Omicron sequence [26]. The final modeled structure was prepared, cleared and minimized.

### 2.2. SANCDB Screening for Drug-Like Molecules

Prior to the structure-based molecular search for potent drugs, the whole SANCDB (https://sancdb.rubi.ru.ac.za/) (accessed on 10 January 2022) was filtered for drugs-like molecules that obey Lipinski’s rule of five [27]. The whole database was filtered for toxicophores, drug-like molecules, and the removal of pan assay interference (PAINS) compounds, and Eli Lilly MedChem rules was used together with the FAF-Drugs4 server (https://fafdrugs4.rpbs.univ-paris-diderot.fr/) (accessed on 10 January 2022) to filter drug-like molecules which are non-toxic, and non-PAINS compounds [28]. The shortlisted compounds were submitted to PyRx for ligand preparation and used the MM2 force field for energy minimization [29].

### 2.3. Computational Molecular Screening of the SANCDB

The virtual screening of compounds against the interface residues of the spike protein in the RBD (449, 453, 455, 456, 486, 487, 489, 493, 496, 498, 500, 501, 502, and 505) was carried out by using the PyRx virtual screening tool [29]. The grid box size of 6.04 × −70.97 × 24.028 was generated by selecting the above-mentioned residues, which were experimentally identified in previous studies for defining the active site in order to bind the ligand. The SANCDB was screened by using a three-step approach. In the first step, the PyRx was used to screen the whole database using an exhaustiveness value of 16, while in the second step the shortlisted top hits were screened again with 32 exhaustiveness. Finally, for the highest-scoring compounds, an exhaustiveness value of 64 was set using the induced-fit docking (IFD) approach to validate the final hits. For the IFD, the AutoDockFR software was used which increased the success rate of docking by optimizing the conformation of receptor sidechains [30].

### 2.4. Protein–Ligand Complexes MD Simulation

The final hits shortlisted from the SANCDB were subjected to molecular dynamics simulation using the AMBER20 package by recruiting the ff19SB and the general AMBER force field (GAFF) that were used for the parameterization of the RBD of the spike protein of the Omicron variant and ligand, respectively [31,32]. An antechamber module was used for drug topologies, and an OPC (Optimal Point Charge) water box was added for the solvation of each protein–ligand complex. Finally, the system was neutralized by counter ions (Na^+^ and Cl^−^). Afterwards, the two-stage energy minimization system (steepest descent and conjugate gradient) was used and followed by the equilibration and heating steps. Finally, a 100 ns MD simulation was completed for the protein–ligand complexes. To treat the long-range electrostatics interactions with a 10.0 Å cutoff distance, the Particle mesh Ewald (PME) algorithm was used. However, the covalent bonds, if any, were treated with the SHAKE algorithm. Finally, the CPPTRAJ package was used to analyze the trajectories, while the PMEMD.cuda was used for running the simulations [33].

### 2.5. Post-Simulation Validation of the Top Hits

#### 2.5.1. The Binding Free Energy Calculations

For the estimation of the binding free energies of various biological complexes such as protein–DNA/RNA or protein–protein, the MMGBSA is the most authentic approach used in different research investigations [34,35,36,37,38]. In the present study, the script MMPBSA.py was used to calculate the binding free energy of the protein–ligand complexes by considering 2500 snapshots.

The following equation was used for binding free energy estimation:

ΔGbind=ΔGcomplex−[ΔGreceptor+ΔGligand],

where ΔG_*bind*_ represents the total binding energy, while ΔG_*receptor*_, ΔG_*ligand*_, and ΔG_*complex*_ represent the binding energies of the protein, the drug, and the complex, respectively. The following equation was used to estimate the individual binding energies such as bonded (G*bond*), electrostatic (G*ele*), polar (G*pol*), and non-polar (G*npol*) energies, which contributed to the total binding free energy (TBFE):

G=Gbond+Gele+GvdW+Gpol+Gnpol.

#### 2.5.2. Prediction of Bioactivity of the Top Hits

To analyze the IC_50_ (Inhibitory Concentration) value for each shortlisted compound, a cheminformatics tool, Molinspiration (https://www.molinspiration.com/cgi-bin/properties) (accessed on 12 January 2022), was used. Molinspiration is used in thousands of research to predict bioactivity scores [39,40,41,42,43].

#### 2.5.3. Determination of K_D_ (Dissociation Constant)

Furthermore, the PRODIGY (PROtein binding enerGY) online server (https://wenmr.science.uu.nl/prodigy/) (accessed on 12 January 2022) was used to identify the dissociation constant values for various biological complexes in order to provide the persuasive knowledge of the dissociation constant (K_D_) [44,45].

## 3. Results and Discussion

### 3.1. Structural Modeling and Screening

**Figure 2:**
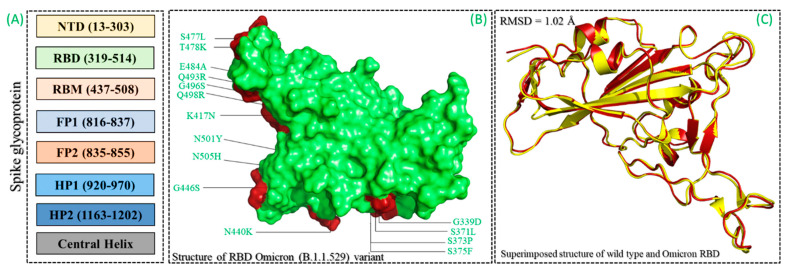
(**A**) Domains organization of spike glycoprotein. (**B**) Structure of reception-binding domain (RBD) and mutations mapping reported in the Omicron variant (B.1.1.529). (**C**) Superimposed structures of the wild-type and B.1.1.529 RBDs.

The RDB of the Omicron (B.1.1.529) variant was modeled harboring 15 substitutions (G339D, S371L, S373P, S375F, K471N, N440K, G446S, S477N, T478K, E484A, Q493R, G496S, Q498R, N501I, and Y505H) using Modeler modeling software. The spike glycoprotein was an important determinant of infection initiation, which led to severe disease outcomes (Figure 2A–C). The binding differences induced by these mutations were linked with immune evasion, thus demanding a further investigation to design a potent drug molecule that can inhibit all the variants [46]. Herein, computational molecular screening and simulation approaches were used to identify novel potent drugs from medicinal plants sources by screening the SANCDB. The interface residues of the RBD were selected as the targets for screening. Prior to whole database screening, Lipinski’s rule of five was applied to filter drug-like molecules and obey the R5 rules. The initial filtration filtered out 1032 compounds of the total 1245 compounds selected. A total of 213 compounds were excluded because of the R5 rules violation. A multi-step screening of the 1032 compounds was performed. In the first step, all the compounds were screened, which resulted in docking scores ranging from −6.8 to −5.2 kcal/mol. A threshold of −5.0 kcal/mol was set to select the top hits from the 1032 molecules screened against the RBD. Among the total, only 418 compounds were selected for the second round of screening. The IFD approach was then employed to screen the top 418 compounds, which resulted in docking scores ranging from −9.63 to −6.71 kcal/mol. Among these, only the top 10 were selected for the docking using Auto Dock Vina. Among the 42 compounds (10%), only 11 compounds were reported to have the highest scores with good interaction profiles. Among the 11 compounds, SANC00944, SANC01032, SANC00992, and SANC00317 were selected for further analysis. The top four compounds were selected for detailed investigation and are given in Table 1.

### 3.2. Binding Mode of 1,2,3,6-Tetragalloylglucose (SANC00944)

*1,2,3,6-Tetragalloylglucose* was originally isolated from *Ceratonia siliqua* and has been reported to exhibit potential anti-oxidative, anticholinesterase, and anti-fungal activity [47]. Herein, the anti-viral activities of these molecules were reported by targeting the RBD of the spike protein from SARS-CoV-2. Docking against the Omicron variant (B.1.1.529) reported a score of −9.35 kcal/mol with three hydrophobic interactions established by Tyr453, Leu455, and Phe456. On the other hand, eight hydrogen bonds were reported in this complex. The targeted amino acids included Arg403, Glu406, Asn417, Tyr449, Tyr453, and Ser496. Arg403 and Ser496 formed two hydrogen bonds each, while the rest established only a single hydrogen bond.

**Figure 3:**
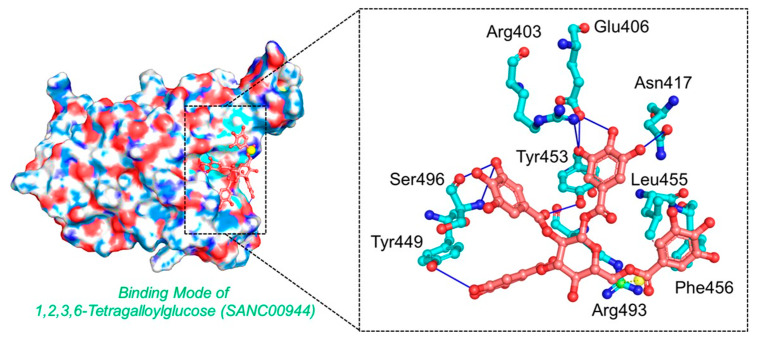
The interaction pattern of *1,2,3,6-Tetragalloylglucose* (SANC00944). The left panel shows the surface representation and the binding conformation of *1,2,3,6-Tetragalloylglucose* (SANC00944), while the right panel show the 3-dimensional interaction pattern.

The only salt bridge was established by Arg493aa, which has been previously reported to play an important role in the anchor locking mechanism and the tighter binding of the RBD to the host receptor hACE2 [12]. Moreover, this compound also targets mutated residues such as Asn417 and Ser496, which shows the potential of this compound against the Omicron and other variants because some of the important mutations are already harbored by the other reported variants. The interaction pattern of *1,2,3,6-Tetragalloylglucose* (SANC00944) is shown in Figure 3. The left panel shows the surface representation and the binding conformation of *1,2,3,6-Tetragalloylglucose* (SANC00944), while the right panel shows the 3D interaction pattern.

### 3.3. Binding Mode of Amentoflavone (SANC01032)

**Figure 4:**
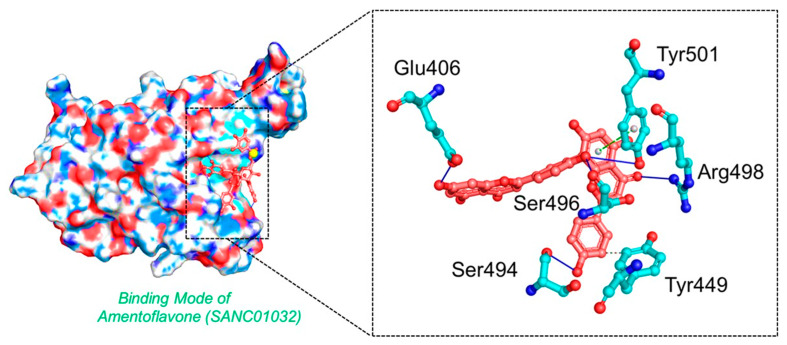
The interaction pattern of *Amentoflavone* (*SANC01032*). The left panel shows the surface representation and the binding conformation of *Amentoflavone* (SANC01032), while the right panel shows the 3-dimensional interaction pattern.

Amentoflavone is a flavone from *Struthiola argentea*, which has been reported to have anti-helminthic activity against many parasites [48]. This molecule established a single hydrophobic interaction formed by Tyr449 and five hydrogen bonds including Glu406, Ser494, Ser496, Arg498, and Tyr501 with a docking score of −8.41 kcal/mol. It can be seen that most residues blocked by this molecule are newly mutated, thus verifying the efficacy of this drug against the Omicron variant. Moreover, Tyr501 also established a π-stacking interaction, which is the most important residue and is linked with higher transmissibility and infectivity in other variants. The interaction pattern of *Amentoflavone* (SANC01032) is shown in Figure 4.

### 3.4. Binding Modes of Luteolin (SANC00992) and Quercetin (SANC00317)

**Figure 5:**
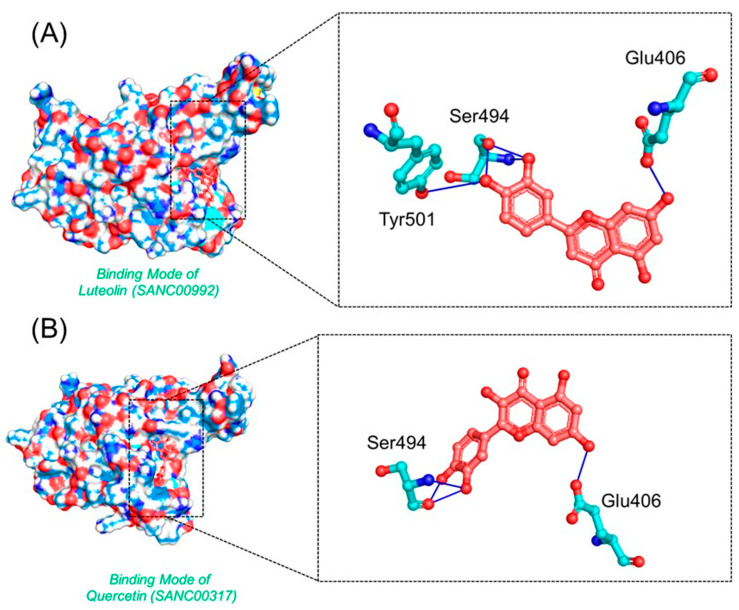
The interaction patterns of *Luteolin (SANC00992)* and *Quercetin (SANC00317)*: (**A**) the binding of *Luteolin* (SANC00992); (**B**) the 3-dimensional interaction pattern of *Quercetin (SANC00317)*.

*Luteolin* and *Quercetin* exhibit similar scaffolds and have been reported to have anti-microbial, anti-cancerous, and anti-SARS activities [49,50,51]. The *Luteolin* compound reported five hydrogen bonds by targeting the three important residues Glu406, Ser494, and Tyr501. Quercetin, on the other hand, established five hydrogen bonds with only two residues, Glu406 and Ser494. The docking score for these two compounds was reported to be −6.99 and −6.93 kcal/mol, respectively. The interaction patterns of *Luteolin* (SANC00992) and *Quercetin* (SANC00317) are shown in Figure 5A,B, respectively.

### 3.5. Dynamic Stability of the Top Hits

**Figure 6:**
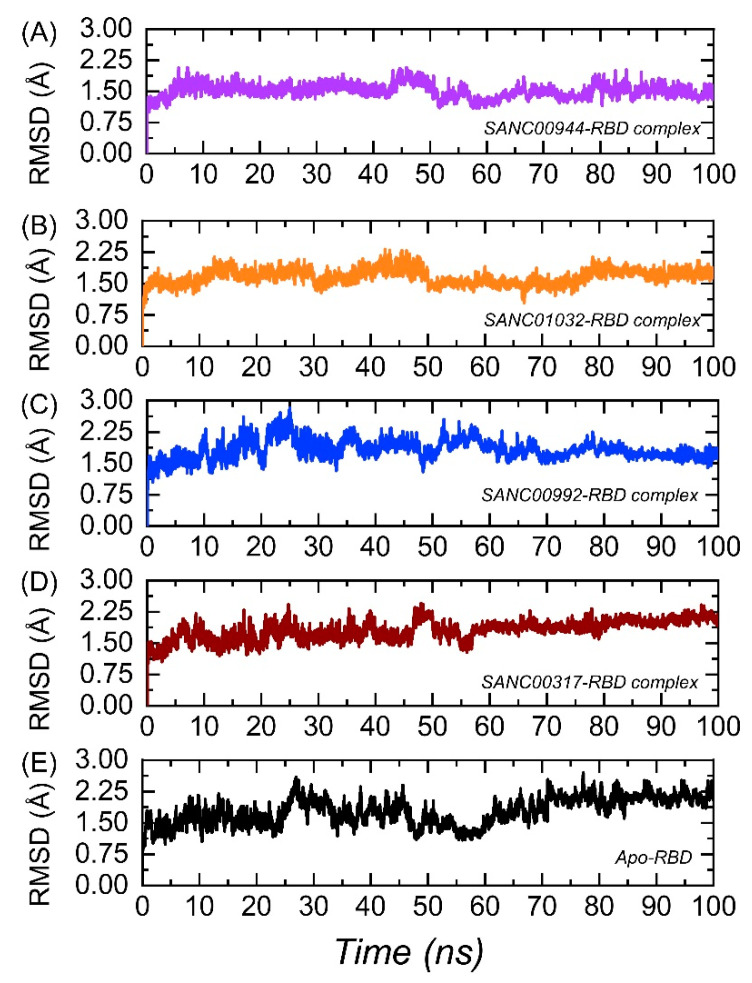
Dynamic stability analysis of the top hits in complex with the RBD: (**A**) the root-mean-square deviation (RMSD) of the SANC00944–RBD complex; (**B**) the RMSD of the SANC01032–RBD complex; (**C**) the RMSD of the SANC00992–RBD complex; (**D**) the RMSD of the SANC00317–RBD complex; (**E**) the RMSD for the Apo RBD.

**Figure 7:**
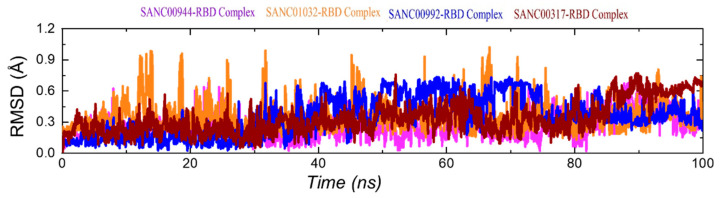
RMSDs of the bound ligands only during the simulation.

The calculation of the dynamic stability within the binding cavity is an important parameter to estimate the binding stability of a ligand inside the pocket. It is important to estimate the binding stability to deliver information about the inhibition of a particular protein steered by different kinds of interactions. Thus, to observe the dynamic stability of each complex over the simulation time period, root-mean-square deviation (RMSD) was computed using CPPTRAJ. The RMSD(s) graphs of the complexes and the Apo are given in Figure 6A–E. It can be seen that the SANC00944–RBD complex demonstrated a dynamically stable behavior during the simulation (Figure 6A). No significant structural perturbation was experienced by the system. The structure demonstrated a rigid dynamic behavior by reaching the equilibrium at 3 ns and the stability at 1.50 Å. The RMSD was further stabilized and did not report any significant deviation from 50 to 100 ns. The average RMSD for the SANC00944–RBD complex was estimated to be 1.50 Å. Hence, this showed that the binding of SANC00944 on the binding interface of the RBD was more stable and produced the inhibitory potential more efficiently. Moreover, the SANC0132–RBD complex also demonstrated a similar behavior to the SANC00944–RBD complex. An equivalent pattern in the RMSD graphs of the SANC00944–RBD and SANC01032–RBD complexes can be seen with the exception of a minor decrease in the RMSD after 30 ns for the SANC01032–RBD complex. The structure reported a uniform RMSD after 50 ns, except a smaller acceptable deviation between 65 and 75 ns. The average RMSD for the SANC01032–RBD complex was reported to be 1.48 Å (Figure 6B). Unlike the SANC00944–RBD and SANC01032–RBD complexes, the SANC00992–RBD complex reported a little unstable behavior comparatively. The RMSD initially remained uniform between 1 and 10 ns; however, the RMSD then increased for a shorter period (11–18 ns). A small decrease in the RMSD for 2–3 ns (18–21 ns) was then experienced, and then, the RMSD increased and remained higher until 26 ns. The complex then was equilibrated and reported a stable dynamic for the rest of the simulation time period although minor deviations at different time intervals were observed. The structure demonstrated a little unstable stable behavior between 51 and 65 ns and then continued to follow the stability trend by showing a stable behavior. The average RMSD of the SANC00992–RBD complex was calculated to be 1.86Å (Figure 6C). In addition, the SANC00317–RBD complex reported a little unstable behavior than the SANC00944–RBD and SANC01032–RBD complexes but comparatively a stable behavior than the SANC00992–RBD complex. This complex also demonstrated initially a stable RMSD until 10 ns and then reported small deviations at different time intervals until 50 ns. With a gradual increase in the RMSD, no perturbation was experienced by the complex until 100 ns. The complex reported an average RMSD of 1.55 Å (Figure 6D). Furthermore, we also calculated the dynamic stability for the Apo state, which revealed that the complexes were stabilized by the binding when compared with the Apo RBD. The Apo RBD as shown in Figure 6E demonstrated a higher RMSD at different time intervals, particularly between 20 and 70 ns, where a significant structural perturbation was recorded. This showed that binding of these drugs stabilized the RBD, thus producing an inhibitory potential and abrogating the binding with ACE2. Furthermore, we also calculated the ligand RMSD for each complex to predict the ligand binding stability. As shown in Figure 7, the average RMSD for each complex was less than 0.8 Å, which demonstrated the stable binding of each ligand during the simulation.

### 3.6. Structural Compactness Analysis of the Top Hits

**Figure 8:**
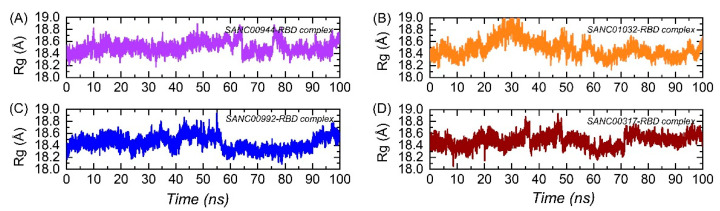
Structural compactness analysis of the top hits in complex with the RBD: (**A**) the Rg of the SANC00944–RBD complex; (**B**) the Rg of the SANC01032–RBD complex; (**C**) the Rg of the SANC00992–RBD complex; (**D**) the Rg of the SANC00317–RBD complex.

The structural compactness of the binding complexes within the binding pocket was estimated to reveal the binding and unbinding events that happened during the simulation. It is a crucial parameter to explore the strength delivered by the bonds upon the binding of a ligand or protein. In this study, structural compactness was calculated as the radius of gyration (Rg) over the simulation time. Each complex here demonstrated the same pattern of Rg as the RMSD. It can be seen that the two complexes, i.e., SANC00944–RBD and SANC01032–RBD complexes, reported a more compact behavior, thus justifying the stable binding of the ligand in the cavity. The average Rg(s) for the SANC00944–RBD and SANC01032–RBD complexes were calculated to be 18.45 and 18.6 Å, respectively. On the other hand, the two complexes, i.e., SANC00992–RBD and SANC00317–RBD complexes, demonstrated a similar pattern of Rg(s) with increased and decreased Rg values at different time intervals. The average Rg(s) for the SANC00992–RBD and SANC00317–RBD complexes were calculated to be 18.72 and 18.68 Å, respectively. The Rg(s) graphs of the complexes are given in Figure 8A–D.

### 3.7. Residual Flexibility Analysis

**Figure 9:**
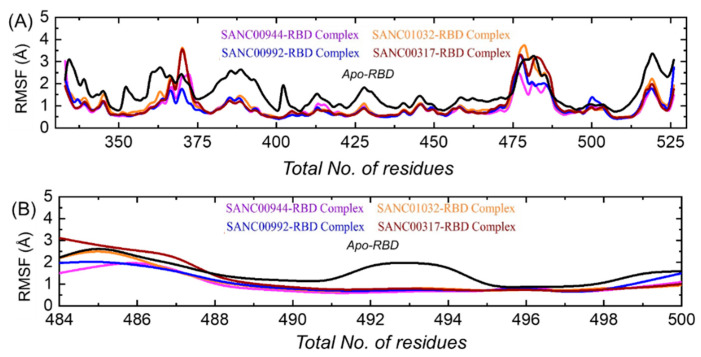
(**A**) Residual flexibility analysis of the SANC01032–RBD, SANC01032–RBD, SANC00992–RBD, and SANC00317–RBD complexes and Apo RBD. (**B**) Flexibility indices of the three binding loops (484–500) required for interactions.

An insight into the residue level fluctuations of the wild-type and the variants was further accomplished, as such a local-level flexibility confers a strength to intermolecular binding, negatively impacts molecular recognition and can potentially influence the overall function of the biological molecule. Herein, the residual flexibility was calculated as root-mean-square fluctuation (RMSF). Higher and lower RMSFs implied flexible and stable regions, respectively (Figure 9A). All of the complexes here demonstrated a similar pattern of the residual flexibility. On the other hand, the Apo RBD demonstrated variations in the RBD residues flexibility. It can be seen that the Apo RBD exhibited a higher flexibility than the drugs-bound complexes. In each complex, the total numbers of residues ranging between 365 and 375 and between 475 and 485 demonstrated a higher flexibility comparatively. The total number of residues ranging 518–525 also reported a higher fluctuation. Previously, the three loops shown in Figure 9B were reported to confer an important role in the higher affinity and the increased transmissibility. In contrast, these residues reported a higher flexibility than the three holo complexes. Abbas et al. reported that the three loops show a higher flexibility in almost all the variants, thus explaining the importance of these loops in the binding characteristics [12,13,15]. To see the flexibility index of these three loops in each complex, the RMSF was also calculated for these loops. It can be seen from Figure 9B that the flexibility of the three loops was reduced by the binding of these drugs, thus inducing inhibitory effects and abrogating the binding with the hACE2 receptor.

### 3.8. Hydrogen Bonding Analysis

**Figure 10:**
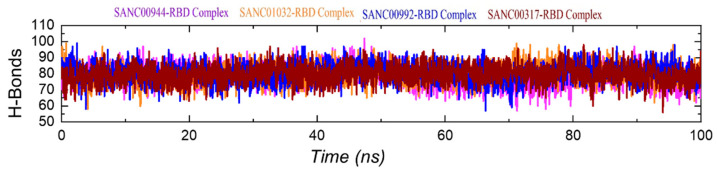
Hydrogen bonding analysis of the SANC01032–RBD, SANC01032–RBD, SANC00992–RBD, and SANC00317–RBD complexes.

The estimation of hydrogen bonds for a protein–ligand complex demonstrates the binding stability and strength of the interacting molecules. Thus, to see the hydrogen bonds during the simulation, we calculated the total number of hydrogen bonds during the simulation. The graph given in Figure 10 shows the hydrogen bonds pattern over the simulation time. In each complex, i.e., SANC01032–RBD, SANC01032–RBD, SANC00992–RBD, and SANC00317–RBD, the average numbers of hydrogen bonds were 85, 84, 85, and 83, respectively. This showed the stable bindings of these compounds. Moreover, for the important residues, the bonding distances were calculated and are shown in Table 2.

### 3.9. Binding Free Energy Estimation

The estimation of small molecules binding free energy using the MM–GBSA approach is the most widely employed method to re-examine the docking orientation, predicting structural stability and binding affinities. The method mentioned above is comparatively less expensive than the extensive alchemical free energy approach. It is also classified as an accurate approach than the rational scoring functions [52]. Considering the high applicability of this approach, we also employed the same approach to estimate the binding free energies for the SANC01032–RBD, SANC01032–RBD, and SANC00992–RBD, and SANC00317–RBD complexes. The TBFE for the SANC01032–RBD complex was reported to be −46.54 kcal/mol; for the SANC01032–RBD complex, the TBFE was −41.88 kcal/mol; for the SANC00992–RBD complex the TBFE was −29.05 kcal/mol, while for the SANC00317–RBD complex the TBFE was −31.03 kcal/mol. The vdWs (Van Der Waal) for the SANC01032–RBD, SANC01032–RBD, SANC00992–RBD, and SANC00317–RBD complexes were reported to be −48.39, −41.27, −36.41, and −32.28 kcal/mol respectively. For these complexes, the respective electrostatic energies were reported to be −18.35, −17.66, −10.39, and −14.23 kcal/mol. This showed that these ligands potently binded to the interface residues and could inhibit the interaction with the hACE2 receptor. The binding free energy results are given in Table 3.

### 3.10. Bioactivity Prediction and Dissociation Constant (K_D_)

**Figure 11:**
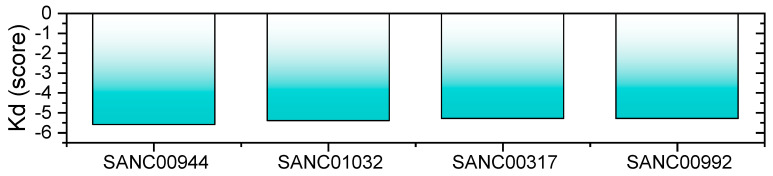
Dissociation constant (K_D_) predicted by prodigy-LIG for the SANC00944–RBD, SANC01032–RBD, SANC00992–RBD, and SANC00317–RBD complexes.

In in silico bioactivity, prediction is a widely used practice for the estimation of IC_50_ values for different classes of druggable proteins. Herein, the molinspiration server predicted bioactivity scores for the SANC01032–RBD, SANC01032–RBD, SANC00992–RBD, and SANC00317–RBD complexes were 0.41, 0.37, 0.25, and 0.22, respectively, which demonstrated a stronger activity against the target protein. The dissociation constant, on the other hand, also re-ranked the final hits and validated the inhibitory potential of these final compounds. The binding constant is a particular case of general equilibrium constants, which measures the bonding affinity between two or more molecules at equilibrium. Understanding binding affinity is key to the appreciation of the intermolecular interactions driving biological processes, structural biology, and structure–function relationships. It is also measured as part of the drug discovery process to help design drugs that bind their targets selectively and specifically. The smaller the K_D_ value, the greater the binding affinity of the ligand for its target. The larger the K_D_ value, the more weakly the target molecule and ligand are attracted to and bind to one another. Binding affinity is influenced by non-covalent intermolecular interactions such as hydrogen bonding, electrostatic interactions, hydrophobic forces, and van der Waals forces between the two molecules. In addition, the binding affinity between a ligand and its target molecule may be affected by the presence of other molecules. The prodigy-LIG server predicted the K_D_ for each complex as −5.6 for the SANC00944–RBD complex, −5.4 for the SANC01032–RBD complex, for the SANC00992–RBD complex −4.8, and −5.3 for the SANC00317-RBD complex. The results showed the inhibitory potential of these compounds by targeting the RBD. The dissociation constant results are shown in Figure 11.

## 4. Conclusions

The current study using a computational molecular search and simulation tools identified several compounds as potential inhibitors of the SARS-CoV-2 Omicron variant (B.1.1.529). Using the multi-step screening approach, the RBD of the spike protein was the target, which revealed SANC00944, SANC01032, SANC00992, and SANC00317 drugs as possible inhibitors of the RBD–ACE2 complex. Post-simulation investigations such as binding free energy calculation, in silico bioactivity, and dissociation constant prediction confirmed the potency of these compounds. In conclusion, this study provides a basis for drug design against the SARS-CoV-2 variants.

## Figures and Tables

**Table 1:** Top four hits identified through the molecular search. The table shows the 2-dimensional structures of the tope hits, their IDs, interactions, and docking scores.

Compound 2D Structure	Compound ID	Interactions	Docking Score (kcal/mol)
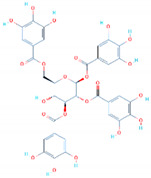	SANC00944	Tyr453, Leu455, Phe456, Arg403, Glu406, Glu406, Asn417, Tyr449, Tyr453, Arg493, Ser496, Ser496,	−9.35
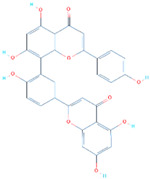	SANC01032	Tyr449, Glu406, Ser494, Ser496, Arg498, Tyr501	−8.41
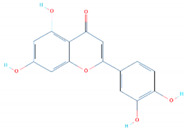	SANC00992	Glu406, Ser496, Ser496, Ser496, Tyr501	−6.99
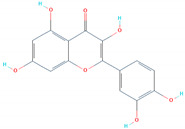	SANC00317	Glu406, Ser496, Ser496, Ser496	−6.93

**Table 2:** Distances of key interacting residues during the simulation.

Complexes Name	Glu406	Asn417	Ser446	Ser494	Arg498	Asn501
SANC00944–RBD	1.8 Å	2.3 Å	3.1 Å	2.96 Å	3.45 Å	2.56 Å
SANC01032–RBD	2.16 Å	-	-	2.46 Å	3.52 Å	-
SANC00992–RBD	2.03 Å	2.14 Å	-	2.88 Å	-	-
SANC00317–RBD	2.93 Å	-	3.58 Å	2.64 Å	-	-

**Table 3:** MM/GBSA results for all the complexes including vdW (Van Der Waal), electrostatic energy, and the total binding energy. All the energies are given in kcal/mol.

Complexes Name	vdW	Electrostatic	SA	GB	Total
SANC00944–RBD	−48.39	−18.35	−11.32	31.52	−46.54
SANC01032–RBD	−41.27	−17.66	−8.24	25.29	−41.88
SANC00992–RBD	−36.41	−10.39	−10.02	27.77	−29.05
SANC00317–RBD	−32.28	−14.23	−9.66	25.14	−31.03

## Data Availability

The data presented in this study are available within the article.
